# Self-treatment of psychiatric conditions using ketamine: Patterns, characteristics, and retrospective insights

**DOI:** 10.1177/02698811251378509

**Published:** 2025-10-22

**Authors:** Gabrielle Smith, Timothy Piatkowski, Jason Ferris, Benjamin Bonenti, Emma Davies, Monica J. Barratt, Celia Morgan, Adam Winstock, Cheneal Puljević

**Affiliations:** 1 School of Public Health, The University of Queensland, Brisbane, QLD, Australia; 2 School of Applied Psychology and Centre for Mental Health, Griffith University, Brisbane, QLD, Australia; 3 Centre for Health Services Research, The University of Queensland, Brisbane, QLD, Australia; 4 Centre for Psychological Research, Oxford Brookes University, UK; 5 Social Equity Research Centre and Digital Ethnography Research Centre, RMIT University, Melbourne, VIC, Australia; 6 National Drug and Alcohol Research Centre, UNSW Sydney, NSW, Australia; 7 Department of Psychology, University of Exeter, UK; 8 University College London, UK; 9 Global Drug Survey, London, UK

**Keywords:** drugs, harm reduction, mental health, self-treatment, ketamine

## Abstract

**Background:**

While research on novel therapeutic applications of ketamine is expanding, particularly in controlled settings, there is limited exploration of its consumption related to self-treatment of psychiatric conditions. This study investigated the characteristics of people who use ketamine and psychedelics for self-treatment of psychiatric conditions, providing insight into patterns of use within this population.

**Methods:**

Utilising the 2020 Global Drug Survey, the analysis incorporates data from 5831 respondents who reported self-treating with unregulated drugs to treat diagnosed psychiatric conditions. We compare three groups: those self-treating with only ketamine (*n* = 242), ketamine and other psychedelics (*n* = 1072), and non-ketamine psychedelic only substances (*n* = 4517). Negative binomial regression was conducted to assess the impact of self-treating psychiatric conditions with ketamine and other psychedelics on the volume of recreational ketamine use.

**Results:**

A high proportion (>60%) had prior psychiatric diagnoses, with depression and anxiety being the most common. People who used both ketamine and other substances reported higher festival and clubbing attendance than the other two groups. People who used ketamine and combined it with other psychedelics used it more frequently (incidence rate ratio (IRR): 0.729, 95% confidence interval (CI): 0.336–1.581), while those using non-ketamine psychedelics only showed a significant reduction in ketamine usage volume (IRR: 0.160, 95% CI: 0.079–0.322) compared to other groups. Almost half of the respondents sought online advice before starting ketamine self-treatment.

**Conclusion:**

This study extends knowledge about various populations using ketamine for self-treatment purposes, proposes areas for future research and suggests online platforms as the most effective place for harm reduction resources relating to ketamine use.

## Introduction

Psychiatric conditions are increasingly prevalent and frequently co-occur with substance use. Globally, one in seven individuals has been diagnosed with at least one mental health and/or substance use disorder (Ritchie and Roser, 2018), and one in three will experience such disorders in their lifetime (Christensen et al., 2020). As these disorders rise, they significantly strain both individuals and healthcare systems, exacerbated by global crises like climate change and the COVID-19 pandemic (Lass-Hennemann et al., 2024; Nersesjan et al., 2023; Walinski et al., 2023). Traditional treatments, including cognitive-behavioural therapy and pharmacotherapy, often fall short due to high relapse rates and adverse effects (Scangos et al., 2023). In this context, emerging treatments like psychedelics are gaining attention, used in both formal and informal treatment settings (Belouin and Henningfield, 2018; Bornemann et al., 2021; Carhart-Harris et al., 2018; Curtis et al., 2020; Ko et al., 2023; Petranker et al., 2022; Rougemont-Bücking et al., 2019; Walker et al., 2024). There are growing reports of people utilising these substances for self-treatment of psychological distress and psychiatric conditions (Walker et al., 2024). While psychedelic substances like psilocybin and LSD dominate headlines, ketamine—a dissociative anaesthetic—has gained attention for its rapid antidepressant effects. It is sometimes grouped within the broader “psychedelic renaissance” despite being pharmacologically distinct (Özgen and van den Brink, 2022; Veraart et al., 2018).

One of the three treatment branches for depression, pharmacologic therapy, involves the prescription of antidepressant medication, which acts upon the monoaminergic system, but it can take several weeks to months to provide a clinical response (Ciraulo et al., 2011; Yavi et al., 2021). However, research indicates that monoaminergic therapies do not guarantee symptom remission for all individuals with major depressive disorder, with up to two-thirds failing to achieve remission, and over 30% potentially meeting criteria for treatment resistance (Karrouri et al., 2021; Vasiliu, 2022). With many of these standard treatments not sufficiently able to manage depression symptoms amid persistently high prevalence rates of mental illness, psychedelic substances have emerged as potential novel psychiatric treatment options (Belouin and Henningfield, 2018; Curtis et al., 2020; Gardner et al., 2019; Nichols et al., 2017). Clinical trials indicate psychedelics hold promise for psychiatric conditions with fewer side effects than traditional medications (Davis et al., 2021; Mitchell et al., 2023). For instance, they show efficacy in treating depression, anxiety, and substance use disorders with lasting effects (Ko et al., 2023; Wolff et al., 2020). Despite regulatory barriers (Berger and Fitzgerald, 2023; Butlen-Ducuing et al., 2023; The Lancet Regional Health-Europe, 2023), their potential is palatable in the extant literature (Gründer and Jungaberle, 2021; Li et al., 2023), though delays in practice may push individuals toward self-exploration (Anderson et al., 2019; Bornemann et al., 2021; Petranker et al., 2022; Žuljević et al., 2023).

Among the currently available psychedelic substances, ketamine is the first potential rapid-acting antidepressant treatment that has robust evidence showing its short-term efficacy in reducing symptoms of depression (Katalinic et al., 2013; Serafini et al., 2014; Singh et al., 2017; Smith-Apeldoorn et al., 2022; Yavi et al., 2022) and suicidal ideation (Abbar et al., 2022; Witt et al., 2020). The past two decades have seen a growing body of evidence to support the use of ketamine as a rapid acting antidepressant treatment option (Grabski et al., 2020; Witt et al., 2020). Given in low sub-anaesthetic doses, it has been shown to effectively treat individuals who were resistant to standard antidepressant treatments, with noted rapid onset of effects (Witt et al., 2020). Ketamine has also consistently shown reductions in suicide ideation (Abbar et al., 2022). Further clinical trials of ketamine use for other psychiatric conditions, such as bipolar disorder, and substance use disorders have also been conducted and show a promising evidence base for its efficacy for these conditions (Bahji et al., 2021; Walsh et al., 2022).

Following the results of these trials, an intranasal esketamine spray was approved by the United States’ Food and Drug Administration (FDA) in 2019 for the treatment of treatment-resistant depression. The FDA approval permits its prescription in conjunction with a newly initiated oral antidepressant, but only for individuals who have failed to respond to at least two prior standard antidepressant treatments. Due to the risks of dissociation, sedation, and potential misuse, intranasal esketamine spray is only available under strict conditions (Kasper et al., 2021). By 2021, both the Australian Therapeutic Goods Administration and the European Commission had also approved esketamine with similar prescribing guidelines (Thornton et al., 2023; Torjesen, 2022). Despite this regulatory progress, ketamine itself remains unapproved for psychiatric indications by the FDA (Pai et al., 2022; Ziegler et al., 2021). Notably, the FDA has explicitly cautioned against the use of compounded ketamine for mental health treatment, citing concerns over inconsistent product quality, lack of rigorous evidence, and serious safety risks—including dissociation, sedation, and abuse potential (Pai et al., 2022; Vekhova et al., 2025; Ziegler et al., 2021). This distinction between esketamine and racemic ketamine is critical when interpreting self-treatment patterns observed outside of formal medical settings (Pai et al., 2022; Vekhova et al., 2025).

In addition to its clinical applications (Hassan et al., 2022), ketamine has become increasingly popular as a recreational substance, particularly in clubbing, rave, festival, and electronic dance music settings (Morgan et al., 2012; Muetzelfeldt et al., 2008; Puljević et al., 2024; Vaz et al., 2022). Its appeal in these contexts is primarily attributed to its euphoric, empathogenic, and sensory-enhancing effects, such as intensifying music and altering perceptions (Moore and Measham, 2008). High levels of use have also been documented among men who have sex with men (Guerras et al., 2021; Kňažek et al., 2023; Melendez-Torres et al., 2018). These hedonistic effects are known to be subject to rapid tolerance, often prompting users to increase their dose to maintain desired effects—escalating into dissociative or hallucinogenic states (Morgan et al., 2012; Muetzelfeldt et al., 2008). While some users may intentionally seek out the dissociative experience, it is more commonly encountered as a secondary consequence of higher-dose use, rather than the primary motivation for recreational consumption (Moore and Measham, 2008; Morgan et al., 2012; Muetzelfeldt et al., 2008).

As ketamine is increasingly consumed recreationally in many countries (Australian Institute of Health and Welfare, 2024; Barrios et al., 2025; Guerrini et al., 2025; Marongiu et al., 2025; Yang et al., 2025), considering its antidepressant effect potential, it appears likely psychopharmacological consumption will also increase (Özgen and van den Brink, 2022). This mirrors trends seen in access to other drugs when conventional prescription-based treatments remain restricted (Piatkowski et al., 2024b). As awareness of ketamine use as an antidepressant increases, an increased understanding of the characteristics of people using ketamine for the self-treatment of psychiatric conditions is needed. Understanding and gaining insight into the experiences of those using ketamine to self-treat psychiatric conditions will assist us to identify different populations who use ketamine, identify their specific therapeutic needs, develop appropriate education and harm reduction resources and gain greater clarity into potential gaps within current psychiatric treatment pathways and services.

This study aimed to examine patterns and characteristics of ketamine use among individuals self-treating psychiatric conditions. We compared three groups: (1) ketamine only; (2) ketamine combined with other psychedelics; and (3) psychedelics excluding ketamine. A secondary aim was to explore differences in total ketamine consumption across these profiles. Finally, we examined whether reported experiences of ketamine self-treatment differed between individuals currently taking prescribed psychiatric medications and those who were not. Given that esketamine prescribing guidelines require concurrent use of a standard antidepressant, we hypothesised that respondents using prescribed medications would report greater perceived effectiveness of ketamine across a range of outcome domains.

## Methods

### Sampling and recruitment

The Global Drug Survey (GDS) is an annual online survey that collects observational cross-sectional data on alcohol and other drug use (Barratt et al., 2017). Eligibility criteria for participating in the GDS include being above 16 years of age and reporting the use of at least one drug in the past 12 months (Winstock et al., 2022). This study utilised data from the 2020 GDS survey, which was completed by 112,341 respondents from 37 countries. The 2020 dataset was selected because it was the most recent publicly available wave at the time of study planning and contained a large, diverse international sample with detailed information on self-treatment practices. Data collection for the 2020 survey ran from 7 November 2019 to 5 February 2020. This study’s sample was restricted to participants who provided a valid answer to a question asking whether they used any of the following substances in the last 12 months with the specific intention of treating their diagnosed psychiatric condition: LSD, magic mushrooms, ketamine, MDMA, peyote, San Pedro, DMT, 5-Meo DMT (toad venom), ayahuasca, kambo and/or ibogaine (*n* = 5831). Although the survey question referred specifically to “diagnosed psychiatric conditions,” diagnoses were self-reported and not clinically verified. It is therefore possible that some participants were self-treating based on perceived or undiagnosed mental health concerns.

### Materials and procedure

Relevant survey items are listed in Supplementary Table 1. Respondents identified which psychedelic substances were used to self-treat psychiatric conditions. Respondents were asked “Which of the following substances have you used in the last 12 months with the specific intention of treating your diagnosed psychiatric condition?” (with the 11 drugs listed above). From these responses, respondents were grouped into one of three categories: those who reported “ketamine use only” to self-treat a psychiatric condition, those who reported using “ketamine plus other psychedelics” to self-treat a psychiatric condition, and those who reported using “psychedelics not including ketamine” to self-treat a psychiatric condition. Survey responses to the frequency and amount of ketamine use (for any purpose) were used to calculate an outcome variable for the total volume of ketamine used (in g) over a year. Covariates selected for inclusion in the model were age, gender, sexual orientation, residential area (urban/regional/remote), clubbing frequency (never/once every 3−12 months/once or twice a month/once a week or more) and number of festivals attended in the past 12 months.

To address the final aim, we analysed a range of survey questions asking respondents regarding the effects and consequences of use of the psychedelic substance that they indicated was most useful for self-treating their psychiatric condition. The sub-sample for this analysis consisted of respondents who indicated that they found ketamine to be the most useful substance to self-treat their psychiatric condition (*n* = 521, 8.9% of the original study sample) and also provided a valid response to whether they reported current use of prescribed psychiatric medication. Results are provided for those who reported use of prescription psychiatric medication (*n* = 168) versus those who did not (*n* = 119).

### Data analysis

Descriptive statistics were calculated to compare socio-demographic characteristics, ketamine use, and psychiatric-related variables for all three groups of respondents reporting use of psychedelics to self-treat a psychiatric condition (ketamine only, ketamine and other psychedelics, psychedelics not including ketamine). Bivariate and multivariate negative binomial regression was used to examine the associations between respondents who reported use of ketamine only, ketamine and other psychedelics and psychedelics not including ketamine for the purpose of self-treating a psychiatric condition and overall ketamine volume use. Age and gender were included as an interaction term to control for known confounding effects on substance use (Barratt et al., 2017). Moreover, based on this prior work, age was modelled as a non-linear term with age being represented by coefficients for both age and gender. Sexual orientation, residential living area, and festival attendance and clubbing frequency were included as covariates in the multivariate model. These covariates were selected because age and gender have known differences relative to substance use (Barratt et al., 2017); it was expected that there are higher rates of ketamine use amongst those with prior experience with the drug (Morgan et al., 2009), common in club, festival, and dance party settings and access to ketamine use is dependent on the availability of the drug, which has been found to be more prevalent in urban settings compared to rural or remote areas (Du et al., 2015). Moreover, given the varying policies, legislations, and community perceptions around ketamine use, analysis was clustered by country to account for potential unmeasured differences. For the negative binomial regression models, analysis was undertaken only with respondents from the identified countries—Australia, Germany, United Kingdom, and United States due to sample size. A *p* value of <0.05 was considered statistically significant in analyses, and complete case analysis was used.

To account for small group sizes and ensure model stability, the following adjustments were made prior to analysis. For analytical purposes, response options to the frequency of clubbing question were collapsed from eight to four categories due to small cell size (from “never/4 times a week” to “never/once every 3−12 months/once or twice a month/once a week or more”). The GDS captures gender as man, woman, non-binary, and different identity. However, for the analytical model, as the representation of non-binary and different identity was expected to be too small for meaningful estimates to be calculated, and these groups were removed for analysis. Additionally, although respondents in this study lived across 37 different countries, due to small counts in particular countries (<5) only the respondents from the top 4 countries (Australia, Germany, United Kingdom, and United States) remained identified with respondents from the remaining country responses being collapsed into the one “Other country” category. The ketamine-only group was retained as the reference category in the regression model as it represents the most isolated self-treatment profile, providing a logical baseline for comparison with poly-substance and non-ketamine self-treatment groups.

The third aim of this paper was to explore responses related to the experiences of psychedelic use, for those who selected ketamine as the most useful substance for self-treating psychiatric conditions, and comparisons for the reported experiences of positive and negative effect outcomes of ketamine use for self-treating psychiatric conditions were drawn between those who currently take prescribed psychiatric medication and those who do not. Participants who reported negative consequences of ketamine use for self-treating psychiatric conditions overall were then asked to rate various negative consequences as none (i.e. did not experience that consequence), mild, moderate, or severe. As such only descriptive statistics have been presented. Analyses were conducted using Stata v16.

### Ethics approval

The 2020 GDS survey received ethical approval from The University of Queensland (2017001452), University College London Research Ethics Committee (Number: 141/02) and The University of New South Wales (HREC HC17769).

## Results

Four thousand eight hundred thirty-one respondents completed the question pertaining to psychedelic use for the purpose of self-treating psychiatric conditions (5.2%). Table 1 provides characteristics of all three groups of respondents reporting use of psychedelics to self-treat a psychiatric condition (ketamine only, ketamine and other psychedelics, psychedelics not including ketamine).

**Table 1.** Sociodemographic characteristics of respondents who reported using psychedelic substances for the self-treatment of psychiatric condition (*n* = 5831).

Characteristics	Self-treating psychiatric condition with only ketamine, *N* = 242		Self-treating psychiatric condition with ketamine and other substances, *N* = 1072		Self-treating psychiatric condition with other substances not including ketamine, *N* = 4517	
	Median	IQR	Median	IQR	Median	IQR
Age	25	22; 31	24	20; 29	23	20; 30
	*N* (%) [missing]					
Gender						
Male	144 (59.50)		743 (69.31)		2950 (65.31)	
Female	90 (37.19)		286 (26.68)		1420 (31.44)	
Non-binary	6 (2.48)		32 (2.99)		118 (2.61)	
Different identity	2 (0.83)		11 (1.03)		29 (0.64)	
Sexual orientation						
Bisexual	58 (23.97)		259 (24.16)		1066 (23.60)	
Heterosexual	136 (56.20)		669 (62.41)		2966 (65.66)	
Homosexual	33 (13.64)		69 (6.44)		194 (4.29)	
Other	11 (4.55)		43 (4.01)		181 (4.01)	
Prefer not to say	2 (0.83)		21 (1.96)		66 (1.46)	
Missing	[2 (0.83)]		[11 (1.03)]		[44 (0.97)]	
Ethnicity						
White	213 (88.02)		901 (84.05)		3612 (79.96)	
Black African/Black Caribbean	1 (0.41)		4 (0.37)		12 (0.27)	
Black American	—		1 (0.09)		28 (0.62)	
South-East Asian	—		11 (1.03)		50 (1.11)	
Asian	—		9 (0.84)		22 (0.49)	
Hispanic/Latino	8 (3.31)		46 (4.29)		353 (7.81)	
Aboriginal/Māori	—		—		16 (0.35)	
Native American	1 (0.41)		4 (0.37)		14 (0.31)	
Mixed	16 (6.61)		66 (6.16)		283 (6.27)	
Other	2 (0.83)		22 (2.05)		94 (2.08)	
Missing	[1 (0.41)]		[8 (0.75)]		[33 (0.73)]	
Country of residence						
Australia	22 (9.09)		131 (12.22)		474 (10.49)	
Germany	62 (25.62)		167 (15.58)		588 (13.02)	
United Kingdom	34 (14.05)		129 (12.03)		256 (5.67)	
United States	46 (19.01)		232 (21.64)		965 (21.36)	
Other countries	78 (32.23)		413 (38.53)		2234 (49.46)	
Residential area						
City/urban	204 (84.30)		808 (75.37)		3273 (72.46)	
Regional	28 (11.57)		192 (17.91)		902 (19.97)	
Rural/remote	9 (3.72)		70 (6.53)		333 (7.37)	
Missing	[1 (0.41)]		[2 (0.19)]		[9 (0.20)]	
Frequency of clubbing in the last 12 months						
Never	53 (21.90)		232 (21.64)		1469 (32.52)	
Once every 3−12 months	69 (28.51)		326 (30.41)		1481 (32.79)	
Once or twice a month	90 (37.19)		349 (33.56)		1164 (25.77)	
Once a week or more	28 (11.57)		145 (13.53)		331 (7.33)	
Missing	[2 (0.83)]		[20 (1.87)]		[72 (1.59)]	
Festivals attended in last 12 months	1	0; 2	1	0; 3	1	0; 2
Missing	[8 (3.31)]		[43 (4.01)]		[172 (3.81)]	
During the last 12 months, on how many days have you used ketamine?	13.5	6; 37.5	10	4; 30	3	1; 10
Missing	[70 (28.93)]		[364 (33.96)]		[3905 (86.45)]	
On a day that you use ketamine how much would you say you normally use? (g)	0.3	0.2; 0.5	0.3	0.2; 0.5	0.2	0.1; 0.4
Missing	[85 (35.12)]		[394 (36.75)]		[3966 (87.80)]	
Total ketamine volume in last 12 months (g)	5	1.6; 16.8	3	1; 10	0.9	0.3;3
Missing	[87 (35.95)]		[396 (36.94)]		[3968 (87.85)]	
	*N* (%) [missing]					
Previous diagnosis with a mental illness						
Yes	156 (64.46)		674 (62.87)		2465 (54.57)	
No	86 (35.54)		396 (36.94)		2048 (45.34)	
Missing			[2 (0.19)]		[4 (0.09)]	
Previous mental illness diagnoses						
Depression	127 (52.48)		540 (50.37)		1929 (42.71)	
Anxiety	101 (41.74)		436 (40.67)		1592 (35.24)	
Bipolar	23 (9.50)		98 (9.14)		319 (7.06)	
Psychosis	8 (3.13)		52 (4.85)		145 (3.21)	
ADHD	41 (16.94)		190 (17.72)		624 (13.81)	
Other	44 (18.18)		144 (13.43)		458 (10.14)	
Missing	[86 (35.54)]		[398 (37.12)]		[2052 (45.43)]	
Main diagnosed psychiatric condition or emotional distress that psychedelics were used to self-treat						
Depression	119 (51.29)		426 (41.11)		1568 (36.54)	
Anxiety	32 (13.79)		180 (17.51)		824 (19.20)	
OCD	2 (0.86)		10 (0.97)		23 (0.54)	
Bipolar	9 (3.88)		23 (2.24)		78 (1.82)	
PTSD	9 (3.88)		51 (4.96)		183 (4.26)	
Psychosis	1 (0.43)		9 (0.88)		19 (0.44)	
Alcohol or other substance use disorder	6 (2.59)		40 (3.89)		164 (3.82)	
Anorexia/bulimia	—		1 (0.10)		30 (0.70)	
Overeating (obesity)	2 (0.86)		3 (0.29)		23 (0.54)	
Cancer-related distress	1 (0.43)		—		9 (0.21)	
Distress associated with another medical disorder	1 (0.43)		20 (1.95)		40 (0.93)	
Psychiatric distress associated with cancer diagnosis	—		2 (0.19)		4 (0.09)	
Treating cancer itself	—		—		1 (0.02)	
To increase appetite	—		—		3 (0.07)	
Bereavement	5 (2.16)		34 (3.31)		106 (2.47)	
Chronic pain	3 (1.29)		7 (0.68)		13 (0.30)	
Trauma	6 (2.59)		53 (5.16)		240 (5.59)	
Relationship problem	20 (8.62)		86 (8.37)		558 (13.00)	
Other	16 (6.90)		83 (8.07)		405 (9.44)	
Missing	[10 (4.13)]		[44 (4.10)		[226 (5.00)]	
Currently prescribed medication to treat mental illness						
Yes	85 (35.12)		256 (23.88)		897 (19.86)	
No	38 (15.70)		256 (23.88)		1025 (22.69)	
Missing	[119 (49.17)]		[560 (52.24)]		[2595 (57.45)]	

IQR: interquartile range; OCD: obsessive compulsive disorder; ADHD: attention-deficit/hyperactivity disorder; PTSD: post-traumatic stress disorder.

The median age for all groups was in the early to mid-20s (24−25 years of age). The percentage of men was highest in all three groups as was those who reported identifying as heterosexual. Compared to the other groups, the ketamine only group had the highest percentage of females (37.2%) and people who identified as homosexual (13.8%). The majority of respondents (⩾80%) in all three groups identified as Caucasian. The most common countries of residence, other than “other countries,” were the United States (*n* = 46, *n* = 232, *n* = 965), and Germany (*n* = 62, *n* = 167, *n* = 588) across the groups who self-treated psychiatric conditions with only ketamine, ketamine and other substances, and other substances not including ketamine, respectively. Most respondents in each group reported living in urban areas, but this was more pronounced in the ketamine only group (84.3%).

In relation to overall ketamine use (for any reason), the group using ketamine only to self-treat psychiatric condition had the highest volume of ketamine use over 12 months (5 g) compared to the group self-treating with ketamine and other psychedelics (3 g) and the group self-treating with psychedelics not including ketamine (0.9 g). Although these individuals did not report using ketamine specifically for self-treatment, some reported ketamine use for other reasons (e.g. recreational use), which accounts for the non-zero consumption observed in this group.

The highest reported diagnosed psychiatric condition for all groups was depression followed by anxiety, and when asked which psychiatric condition participants were mainly trying to self-treat when using psychedelics, all three groups reported depression as the number one reason. This was most pronounced in the ketamine-only group (51.3%, *N* = 119), compared to the ketamine and other psychedelics group (41.1%, *N* = 426), and the psychedelics without ketamine group (36.5%, *N* = 1568).

Table 2 reports the results of the negative binomial regression analysis between the three ketamine use groups for self-treating psychiatric condition groups and total ketamine volume. The Wald test revealed an overall group effect difference that was statistically significant (χ^2^_(2)_ = 28.6, *p* < 0.001), which was driven by differences between the reference group (only using ketamine for self-treatment) and those who used psychedelics not including ketamine to self-treat psychiatric condition. Respondents who did not use ketamine for self-treatment (but did use other psychedelics) had an expected rate ratio of 0.151; or 0.151 times fewer grams of ketamine in the last year compared to those who only used ketamine for self-treating their psychiatric concerns. The results suggest no significant difference between the reference group and people who use ketamine as well as other psychedelics to self-treat their psychiatric concerns. After accounting for any association from the additional covariates included in the multivariate model, the results suggested a similar pattern between the three groups and the total volume of ketamine consumed in the last 12 months.

**Table 2.** Negative binomial regression model of associations, of those who use ketamine, between total yearly ketamine volume and use of ketamine and other psychedelics to self-treat psychiatric condition (clustered by country).

Model	IRR	*p* Value	95% CI
Bivariate model (*n* = 872)			
Ketamine only	Reference		
Ketamine and other psychedelics	0.68	0.200	0.38–1.23
Psychedelics not including ketamine	0.15	<0.001	0.07–0.33
Multivariate model (*n* = 837)^a^			
Ketamine only	Reference		
Ketamine and other psychedelics	0.73	0.424	0.34–1.58
Psychedelics not including ketamine	0.16	<0.001	0.08–0.32

IRR: incidence rate ratio; CI: confidence interval.

a Multivariate model adjusted for age, gender, sexual orientation, residential living area, festival attendance, and clubbing frequency (modelled results presented in Supplemental Table 2).

**Figure 1.**
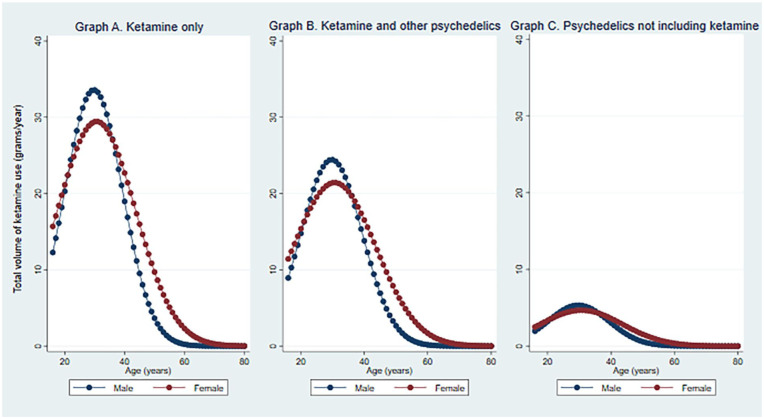
Age and gender predictor margins on total ketamine volume by psychedelic use for self-treating psychiatric condition groups (after adjusting for covariates and clustered by country). See Supplemental Appendix 1 for Figure 1 with confidence bands (these have been excluded here to aid clarity).

Of interest in the multivariate model was the specific association of sex and age on total volume of ketamine consumed in the last 12 months. The interaction term of sex and age (where age has been modelled as a quadratic term) was a significant predictor of total ketamine volume (after adjusting for the other covariates; χ^2^_(3)_ = 222.72, *p* < 0.001). Using Stata’s margins command, Figure 1 depicts the total ketamine volume estimates by sex for each age (in years) of respondents across the three groups. As the representation of non-binary and different identity across the three groups is small. Figure 1 depicts the estimated total ketamine volume only for men and women. As seen in Figure 1, total volume of use—for each sex across each year-age—is greatest for “ketamine only” then ketamine with other psychedelics and then psychedelics not including ketamine. The estimated peak volume of ketamine for males in the ketamine only group is 33.3 g occurring at age 30; the estimated peak volume of ketamine for females in this group is 29.46 g occurring at age 31. The estimated peak volume of ketamine for males in the ketamine and other psychedelics group is 24.0 g occurring at age 30; the estimated peak volume of ketamine for females in this group is 21.26 g occurring at age 31. The estimated peak volume of ketamine for males in the psychedelics not including ketamine group is 5.33 g occurring at age 30; the estimated peak volume of ketamine for females in this group is 4.72 g occurring at age 31. Also notable in Figure 1 is that the estimated total volume of ketamine for females after the age of 40 is higher than for men at the same age.

To address the third aim of this paper, Table 3 provides a summary of use and reported experiences for those who reported ketamine to be the most useful psychedelic for self-treating psychiatric conditions. The average number of days people used ketamine over the last 12 months recreationally was higher than the number of days that they used it for self-treatment purposes for both groups. Respondents reporting not using prescription medicine, reported using ketamine for self-treatment (6.5 days) and recreationally (10 days) less frequently than those reporting prescription medication use (10 and 15 days, respectively).

**Table 3.** Ketamine use behaviours and experiences for those who reported ketamine to be the most useful psychedelic for self-treating psychiatric condition, by those who reported use of prescription psychiatric medication (*n* = 168) versus those who did not (*n* = 119).

Use type	Prescription medicines (*n* = 168)				No prescription medicines (*n* = 119)			
Ketamine use for psychiatric condition (median [IQR])	10 [3–25]				6.5 [3–15]			
Ketamine use for recreational purposes (median [IQR])	15 [5–30]				10 [5–20]			
Effect domain	Prescription medicines (median [IQR])				No prescription medicines (median [IQR])			
Overall psychiatric symptoms	2 [1–2]				2 [1–2]			
Mood/reduced depression	2 [1–3]				2 [1–3]			
Productivity/motivation/confidence	1 [0–2]				1 [0–2]			
Energy/alertness/focus	1 [0–2]				1 [0–2]			
Ability to control negative thoughts/persistent worry	1 [0–2]				2 [1–3]			
Tolerance towards others	1 [0–2]				1 [0–3]			
Feelings of frustration/anger	1 [0–2]				1 [0–2]			
Sight/smell/hearing	0 [0–1]				0 [0–1]			
Understanding why I feel this way	2 [1–3]				2 [1–2]			
Understanding my condition	1 [0–2]				2 [0–3]			
Empathy/sociability/communication	1 [0–2]				1 [0–2]			
Concentration/memory	0 [–1–0]				0 [–1–1]			
Anxiety/social anxiety	1 [0–2]				2 [0–2]			
Sleep	0 [0–1]				0 [0–1]			
Alcohol/drug use	0 [0–2]				1 [0–2]			
Life priorities	0.5 [0–2]				1 [0–2]			
Self-identity	1 [0–2]				1 [0–2]			
Consequence	Prescription medicines (%)				No prescription medicines (%)			
	None	Mild	Moderate	Severe	None	Mild	Moderate	Severe
Disconnection	5.95	16.67	7.74	4.76	6.72	8.40	11.76	6.72
Persistent worrying	14.88	10.12	7.14	2.98	13.45	13.45	1.68	5.04
Comedown/addiction concerns	5.95	13.69	13.69	1.79	6.72	11.76	10.92	5.04
Drug interaction issues	13.69	11.9	8.33	1.19	10.92	11.76	6.72	4.20
Mental confusion	6.55	14.29	10.71	3.57	5.88	13.45	9.24	5.04
Hallucinations	25	7.14	1.79	1.19	20.17	7.56	3.36	2.52
Poor physical health	18.45	9.52	4.17	2.98	14.29	11.76	5.04	2.52
Irrational fears	19.64	5.36	7.74	2.38	13.45	12.62	5.04	2.52
Poor mood/emotion regulation	15.48	12.5	4.17	2.38	14.29	10.92	5.88	2.52
Visual distortions	25	4.17	2.98	2.38	25.21	2.52	2.52	3.36

Effects Domain is measured as a result of ketamine use over the last 12 months on a scale of −3 to +3? (−3 strong negative consequences, 0 no change, +3 strongly positive).

IQR: interquartile range.

Respondents were asked to rate the effects noticed from ketamine use across 17 psychiatric and well-being effects (see Table 3). The results were positive or no change (⩾0) for all effects. Results were similar across both groups (those who do or do not currently use psychiatric medication), with the group not using prescription medicine having some higher—more positive—scores in several effects (“Changes in ability to control negative thoughts/persistent worrying,” “Changes in understanding of my condition or how I relate to it,” “Changes in anxiety, including social anxiety,” “Changes in use of alcohol/other drugs,” and “Changes in life priorities”). For both groups, the effects with the highest reported scores—indicating positively perceived changes—were “Changes in mood or reduced depression,” “Changes in overall symptoms of psychiatric condition,” and “Changes in understanding why I feel the way I do.” In contrast, the effects with the lowest scores—reflecting minimal or no improvement—were “Changes in concentration/memory,” “Changes in sleep” and “Changes in sight/smell or hearing”.

When asked whether they had experienced any negative effects from using ketamine, 34% reported they had. Overall, the group not taking prescription medication had higher percentages of severe responses for all 10 negative consequences (except for “poor physical health”), compared to responses from those taking prescription medication (see Table 3). The outcome with the highest “severe” response from both groups was “Feeling disconnected from the world around you”. Finally, most respondents (>65%) indicated that they obtained advice/information before starting to use ketamine to self-treat. When asked where they sought advice or information before using ketamine for self-treatment, the most popular response was “a website” (44.64%; *N* = 75] for those currently taking prescription psychiatric medication and approximately half (*N* = 57) for those not taking prescription medication). The second most common source was “an online forum” (41.07%; *N* = 69 and 43.70%; *N* = 52), respectively), followed by “a friend/partner/family member” (25%; *N* = 42 and 26.89%; *N* = 32). The least likely source of advice was “a therapist” (3.57%; *N* = 6 and 5.88%; *N* = 7, respectively).

## Discussion

This study aimed to explore ketamine use among people self-treating for psychiatric conditions, comparing those using ketamine alone, ketamine with other psychedelics, and psychedelics excluding ketamine. Additionally, we sought to determine how concurrent use of prescribed psychiatric medications influenced the perceived effectiveness of ketamine for self-treatment. Our findings revealed significant differences in ketamine consumption across these groups of participants, with the highest use among those combining ketamine with other psychedelics. These results highlight diverse self-treatment strategies and underscore the need for targeted harm reduction and clinical guidance.

Our study’s respondents who reported using ketamine for self-treatment tended to report consuming greater amounts of the drug overall. However, not all people who use ketamine recreationally report self-treating psychiatric concerns, highlighting that while overlap exists (Griffiths et al., 2021; Özgen and van den Brink, 2022; Vaz et al., 2022), recreational and therapeutic use are not entirely synonymous. Additionally, our data showed little difference in total ketamine use between those who used it exclusively for self-treatment and those who combined it with other psychedelics, suggesting similar consumption patterns in both groups. Given that ketamine is often used alongside other substances in party settings (Morgan et al., 2009; van Beek et al., 2024), future studies should explore potential interactions or potential effects of using ketamine with other substances in therapeutic contexts. Interestingly, while the median age for ketamine self-treatment was in the early 20s, peak ketamine use across the year both recreational and therapeutic contexts occurred around age 30 for men and women. One possible explanation for this trend is that ketamine’s relatively short duration of effect and minimal long-term consequences (e.g. no hangover) may appeal to older consumers who have more responsibilities or busier lives (Moore and Measham, 2008; Muetzelfeldt et al., 2008). Furthermore, our data revealed that while men exhibited the highest peaks in ketamine use, their total volume of use declined more sharply after age 40 compared to women, suggesting possible gender differences in ketamine consumption patterns later in life. This finding warrants further investigation to determine whether women over 40 have a greater therapeutic need for ketamine, or alternatively, whether reduced use reflects lower perceived effectiveness in this demographic.

We hypothesised that people currently taking prescription psychiatric medications would report better therapeutic outcomes from ketamine use compared to those not on medication. This expectation was based on current esketamine nasal spray prescribing guidelines, which require co-prescription of a new antidepressant after the failure of two prior treatments. Overall, we found that more respondents experienced positive outcomes from therapeutic ketamine use than negative ones, aligning with existing evidence of ketamine’s efficacy as a rapid-acting antidepressant (Daly et al., 2019; Hopwood et al., 2024; Popova et al., 2019; Reif et al., 2023). Notably, respondents not using prescription psychiatric medications reported higher positive outcomes across several domains, including “ability to control negative thoughts/persistent worrying,” “understanding of their condition,” “anxiety reduction,” “reduced alcohol/other drug use,” and “life priorities.” This suggests that the concurrent use of ketamine with prescription psychiatric medications may not offer enhanced benefits over ketamine alone in treating depression. Future research should investigate ketamine’s effects in combination with other psychiatric medications. While our findings suggest that ketamine may offer perceived benefits for some individuals self-treating psychiatric symptoms, the cross-sectional nature of this study limits causal interpretation. Therefore, rather than advocating for earlier introduction of ketamine in treatment pathways, we recommend further longitudinal research to assess its safety, efficacy, and interaction with other medications. For instance, clinicians should remain cautious, particularly in light of existing FDA warnings regarding compounded ketamine formulations used outside approved protocols. Nevertheless, if ketamine proves more effective without requiring conventional antidepressants, many patients could avoid the side effects and withdrawal risks associated with traditional medications. However, when asked to rate the severity of negative consequences experienced from using ketamine to self-treat psychiatric conditions, respondents not on prescription medication were more likely to report certain negative effects from ketamine, despite both groups using similar amounts, including disconnection, mental confusion, and persistent worrying. This underscores the need for more rigorous research into ketamine’s safety, particularly when used with other medications (Hassan et al., 2022; Özgen and van den Brink, 2022). Key gaps remain regarding optimal dosing, side effect management, risk of misuse, and long-term efficacy (Hopwood et al., 2024). Calls for longitudinal studies and standardised databases to track these outcomes are critical to address these uncertainties (Singh et al., 2017).

Lastly, when asked about whether they sought advice or information before starting to self-treat with ketamine, most respondents indicated that they had, further reporting seeking the advice primarily from websites and online forums. This suggests that this population prefers seeking information regarding the use of ketamine from online sources, fitting with extant work specific to ketamine (Chaves et al., 2020; Tackett-Gibson, 2008) as well as other substances (Lamb et al., 2024; Piatkowski et al., 2023). Despite this, we recommend that individuals seek medical supervision as a primary source of guidance, with websites serving as a supplementary avenue for education and harm reduction once professional advice has been received. This includes detailed guidance on dosage, potential side effects, and interactions with other substances. Looking ahead, platforms like the GDS “drug literacy platform,” also aim to address these needs by developing comprehensive harm reduction resources specifically tailored for those using substances like ketamine for self-treatment. Such a platform could serve as a vital tool, bridging the gap between people who use drugs and reliable, evidence-based information. Additionally, resources should be designed to reach diverse audiences, ensuring that information is understandable and actionable for people with varying levels of experience and knowledge. As demonstrating in other drug-using communities such as those who use image and performance-enhancing drugs, investing in person-centred educational content and interactive tools, which adhere to appropriate co-design principles among those who have lived-living experience (Piatkowski et al., 2024c), can further enhance the utility of these resources and their uptake (Piatkowski et al., 2024a). Ultimately, improving the quality and accessibility of online information can facilitate consumers to make informed decisions, reduce harm, and support safer practices in ketamine use.

### Limitations

This study offers novel insights into the experiences of individuals using recreational ketamine for self-treatment of psychiatric conditions. Despite ketamine’s restricted use as an antidepressant, data from a large sample (*n* = 5831) across 37 countries shed light on the behaviours and practices surrounding both recreational and medicinal ketamine use. As one of the few studies addressing this topic, it explores patterns of ketamine use for managing psychiatric conditions in a population often underrepresented in traditional research. However, our findings are subject to some limitations. The GDS relies on a self-recruited online convenience sample of people who use drugs, limiting the ability to generalise findings (Barratt et al., 2017; Winstock et al., 2022); however, these findings set the stage for understanding ketamine use in the community for self-treatment of psychiatric concerns. The cross-sectional nature of the GDS also means that causal relationships and directions between measures are unable to be determined. All responses in the GDS rely upon the respondents’ self-reporting of events over the past 12 months, leaving room for recall bias to occur as well as potentially inaccurate or unreliable reporting of measures. Next, to reduce the survey burden, many questions in the GDS are not compulsory, which results in missing data across many of the variables and restricts the sample able to be included for complete case analysis. Finally, findings related to respondents’ perceived effects and consequences of ketamine use for self-treating psychiatric condition are likely to be biased toward positive outcomes as survey data for these questions were only available for those who had reported that ketamine was the most useful substance for self-treatment purposes. Future studies would benefit from investigating the effects and consequences of ketamine use among those who reported ketamine as not being useful, or those who use ketamine recreationally only.

## Conclusions

This study offers valuable insights into the self-treatment practices of people who use ketamine for psychiatric conditions. The findings demonstrate notable differences in consumption patterns and perceived effectiveness between those using ketamine alone, in combination with other psychedelics, and with prescribed psychiatric medications. While ketamine consumption was generally reported to yield positive therapeutic outcomes, especially among those not on conventional medications, significant negative effects were also observed, particularly among people who used these for self-treatment. These results underscore the complexity of ketamine self-treatment strategies and the need for further research into its safety, efficacy, and interactions with other substances or medications. This research highlights the importance of providing accessible, harm reduction-focused educational resources, particularly via digital platforms, to support safe ketamine use across diverse contexts. Future studies should continue to explore ketamine’s therapeutic potential, with a focus on dosing, long-term outcomes, and optimising its place in the treatment pathway for psychiatric conditions.

## Supplemental Material

sj-docx-1-jop-10.1177_02698811251378509 – Supplemental material for Self-treatment of psychiatric conditions using ketamine: Patterns, characteristics, and retrospective insights Supplemental material, sj-docx-1-jop-10.1177_02698811251378509 for Self-treatment of psychiatric conditions using ketamine: Patterns, characteristics, and retrospective insights by Gabrielle Smith, Timothy Piatkowski, Jason Ferris, Benjamin Bonenti, Emma Davies, Monica J. Barratt, Celia Morgan, Adam Winstock and Cheneal Puljević in Journal of Psychopharmacology

sj-docx-2-jop-10.1177_02698811251378509 – Supplemental material for Self-treatment of psychiatric conditions using ketamine: Patterns, characteristics, and retrospective insights Supplemental material, sj-docx-2-jop-10.1177_02698811251378509 for Self-treatment of psychiatric conditions using ketamine: Patterns, characteristics, and retrospective insights by Gabrielle Smith, Timothy Piatkowski, Jason Ferris, Benjamin Bonenti, Emma Davies, Monica J. Barratt, Celia Morgan, Adam Winstock and Cheneal Puljević in Journal of Psychopharmacology
