# Supplementary material for: Self-treatment of psychiatric conditions using ketamine: Patterns, characteristics, and retrospective insights
Source: J Psychopharmacol. 2025 Oct 22;40(6):933–44. doi: 10.1177/02698811251378509 (PMC13351112; doi:10.1177/02698811251378509)
Supplement: sj-docx-1-jop-10.1177_02698811251378509 – Supplemental material for Self-treatment of psychiatric conditions using ketamine: Patterns, characteristics, and retrospective insights [file sj-docx-1-jop-10.1177_02698811251378509.docx]

**SUPPLEMENTARY TABLE 1.** List of relevant questions and response options from the GDS2020 used in this study

| **Question** | **Response Options** |
| --- | --- |
| How old are you? | *Drop down selection of ages in 1-year increments from 16 – 85, followed by >85* |
| What is your gender? | - Male - Female - Non-binary - Different identity |
| Which country do you currently live in? | *Drop down selection of all countries in alphabetized order* |
| Would you say you are living in a…. | - City/urban area - Regional area - Remote/rural area |
| Your ethnicity? | - White - Black African/ Black Caribbean - Black American - South East Asian (including Chinese, Vietnamese, Japanese, Thai) - Asian (Pakistani, Indian, Bangladeshi) - Hispanic/ Latino - Aboriginal/ Maori - Native American - Mixed - Other (please specify) |
| How often did you go clubbing in the last 12 months? | - Never - Less than once every 3 months - Once every 3 months - Once a month - Once a fortnight - Once or twice a week - 3 to 4 times a week - More than 4 times a week |
| How many festivals have you been to in the last 12 months? | *Free text number* |
| During the last 12 months, on how many days have you used ketamine?  For example: Daily=365, Twice weekly = 104, Weekly = 52, Monthly = 12 | *Free text number* |
| On a day that you use ketamine how much would you say you normally use? (grams) | *Drop down selection* *- 0.1gm increment options from 0.1 – 2.0 gm* *- 0.5gm increment options from 2-5 gm*  *- >5 gm option*  *- don’t know* |
| Have you ever been diagnosed with a mental illness? | - Yes - No |
| Which illnesses have you ever been diagnosed with? | - Depression - Anxiety - Bipolar - Psychosis - ADHD - PTSD - Other (please specify) |
| Are you currently prescribed medication to treat your mental illness? | - Yes - No |
| Which of the following substances have you used in the last 12 months with the specific intention of treating your diagnosed psychiatric condition? | - LSD - Magic mushrooms - Ketamine - MDMA - Peyote - San Pedro - DMT - 5-MEO DMT (toad venom) - Ayahuasca - Kambo - Ibogaine |
| Which diagnosed psychiatric condition or emotional distress were you **mainly** trying to treat when you used these substances? | - Depression - Anxiety - OCD - Bipolar - PTSD - Psychosis - Alcohol or substance use disorder - Anorexia/bulimia - Over eating (obesity) - Cancer-related distress - Distress associated with other medical disorder - Mental health distress associated to cancer diagnosis - Treating cancer itself - To increase appetite - Bereavement - Chronic pain - Trauma - Relationship problem - Other |
| Thinking only of the psychedelic substance you found most useful for self-treating your condition in the last 12 months please tell us: | |
| On how many occasions have you taken this substance in the last 12months for the specific reason of treating your condition?  For example: Daily=365, Twice weekly = 104, Weekly = 52, Monthly = 12 | *Free text number* |
| How many times in the last 12 months did you use this substance purely for recreational purposes?  For example: Daily=365, Twice weekly = 104, Weekly = 52, Monthly = 12 | *Free text number* |
| Which of the following effects have you noticed as a result of your use of this psychedelic substance over the last 12 months on a scale of -3 to +3?  (-3 strong negative consequences, 0 no change, +3 strongly positive, n/a not applicable) For example, if you have a strong decrease in anxiety, you would put +3 for that question. If you had some positive changes in mood/reduced depression you may put in +2 | - Change in overall symptoms of your psychiatric condition - Changes in mood or reduced depression - Changes in productivity, motivation, or confidence - Changes in energy, alertness and/or focus - Changes in ability to control negative thoughts/persistent worrying - Changes in my tolerance towards others - Changes in feelings of frustration / anger - Changes in sight, smell or hearing - Changes in my understanding of why I feel the way I do - Changes in my understanding of my condition or how I relate to it - Changes in empathy, sociability and communication skills - Changes in concentration/memory - Changes in anxiety, including social anxiety - Changes in sleep - Changes in my use of alcohol / other drugs - Changes in life priorities - Changes in self-identity |
| Did you obtain advice/information before you started using this substance for self-treatment? | - Yes - No |
| Where did you obtain this advice/information before you started using this substance for self-treatment? | - A doctor - A therapist - A website - Online forum - Social media/news - A friend/partner/family member - A local psychedelic society - A book - Other |

**SUPPLEMENTARY Table 2.** Extended results to negative binomial regression model of associations with ketamine volume and use of ketamine and other psychedelics to self-treat psychiatric illness with all covariates (clustered by country)

|  | **IRR*** | **P value** | **95% Confidence interval** | |
| --- | --- | --- | --- | --- |
| **Multivariate model (n=837):** | | | | |
| Ketamine only | Reference | | | |
| Ketamine and other psychedelics | 0.729 | 0.424 | 0.336 | 1.581 |
| Psychedelics not including ketamine | 0.160 | <0.001 | 0.079 | 0.322 |
| Male | Reference | | | |
| Female | 6.326 | 0.207 | 0.361 | 110.86 |
| Non-binary | 19.386 | 0.015 | 1.758 | 213.809 |
| Different identity | 0.003 | 0.012 | 0.000 | 0.280 |
| Age | 1.376 | <0.001 | 1.233 | 1.536 |
| gender#c.age: |  |  |  |  |
| Male | Reference | | | |
| Female | 0.870 | 0.155 | 0.719 | 1.054 |
| Non-binary | 0.745 | <0.001 | 0.647 | 0.858 |
| Different identity | 1.377 | 0.224 | 0.822 | 2.305 |
| c.age#c.age | 0.995 | <0.001 | 0.993 | 0.996 |
| gender#c.age#c.age: |  |  |  |  |
| Male | Reference | | | |
| Female | 1.002 | 0.137 | 0.999 | 1.006 |
| Non-binary | 1.005 | 0.000 | 1.004 | 1.007 |
| Different identity | 0.995 | 0.397 | 0.983 | 1.007 |
| Residential living area: |  |  |  |  |
| Urban/city | Reference | | | |
| Regional | 1.117 | 0.637 | 0.706 | 1.766 |
| Remote/ rural | 0.715 | 0.060 | 0.503 | 1.015 |
| Sexual orientation: |  |  |  |  |
| Heterosexual | Reference | | | |
| Bisexual | 1.090 | 0.546 | 0.824 | 1.443 |
| Homosexual | 0.821 | 0.492 | 0.467 | 1.442 |
| Prefer not to say | 1.585 | 0.426 | 0.509 | 4.935 |
| Other | 1.618 | 0.252 | 0.710 | 3.682 |
| Festivals attended (in last 12 m) | 1.063 | <0.001 | 1.037 | 1.090 |
| Clubbing frequency: |  |  |  |  |
| Never | Reference | | | |
| Once every 3-12 months | 1.006 | 0.982 | 0.613 | 1.650 |
| Once or twice a month | 1.040 | 0.936 | 0.400 | 2.705 |
| Once a week or more | 1.614 | 0.075 | 0.952 | 2.738 |
