# Supplementary material for: Self-treatment of psychiatric conditions using ketamine: Patterns, characteristics, and retrospective insights
Source: J Psychopharmacol. 2025 Oct 22;40(6):933–44. doi: 10.1177/02698811251378509 (PMC13351112; doi:10.1177/02698811251378509)

**APPENDIX 1.** Age and gender predictor margins on total ketamine volume by psychedelic use for self-treating psychiatric illness groups (after adjusting for covariates and clustered by country) presented with confidence interval bands


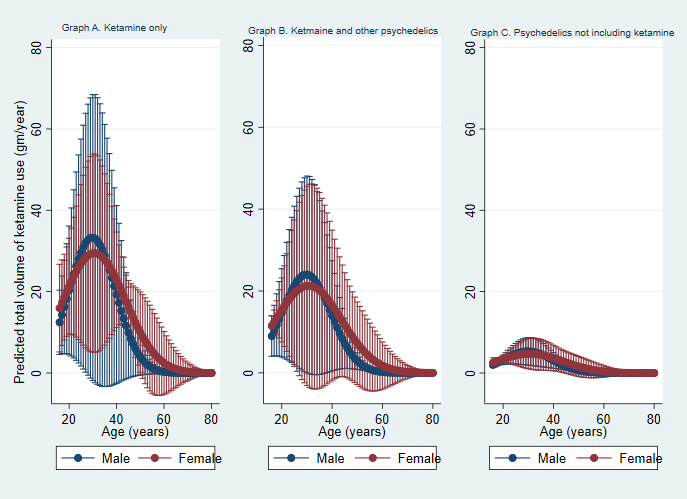

Supplement: sj-docx-2-jop-10.1177_02698811251378509 – Supplemental material for Self-treatment of psychiatric conditions using ketamine: Patterns, characteristics, and retrospective insights [file sj-docx-2-jop-10.1177_02698811251378509.docx]
